# Editorial: Advances in emergency interventional radiology: techniques, outcomes, and future directions

**DOI:** 10.3389/fradi.2026.1986746

**Published:** 2026-09-28

**Authors:** Francesco Tiralongo, Roberto Minici

**Affiliations:** 1 Radiology Unit 1, University Hospital Policlinico “G. Rodolico-San Marco”, Catania, Italy; 2 Radiology Unit, Department of Experimental and Clinical Medicine, Magna Graecia University, Catanzaro, Italy

**Keywords:** damage control interventional radiology, emergency radiology, hemorrhage control, image-guided intervention, interventional management, interventional radiology (IR), transarterial embolization (TAE)

Interventional radiology (IR) has increasingly become an integral component of time-sensitive clinical decision-making. By combining imaging, vascular access, and minimally invasive therapy within the same pathway, IR can control hemorrhage, restore vascular patency, manage procedure-related complications, and preserve organs while limiting the physiological burden of open surgery. The 17 contributions collected in this Research Topic reflect this expanding role across hemorrhagic, neurovascular, oncologic, and percutaneous interventions. Taken together, they also suggest a broader conceptual evolution: IR should increasingly be regarded not merely as a technical service requested after other treatments fail, but as a clinical management strategy that may be definitive, adjunctive, preventive, or temporizing.

This evolution challenges the traditional dichotomy between non-operative management (NOM) and operative management (OM). In trauma guidelines, angiography and angioembolization are commonly incorporated within NOM. WSES guidelines, for example, describe NOM of splenic injury as ranging from observation to angiography and angioembolization, while liver trauma guidelines recommend angiography and angioembolization as a possible first-line intervention in hemodynamically stable patients with arterial blush (1, 2). Yet embolization is neither observation nor conservative therapy. It represents an active, invasive, image-guided treatment with its own indications, timing, technical endpoints, risks, and rescue pathways. Although current nomenclature often retains embolization within NOM, its functional position in many clinical algorithms lies between observation and surgery.

We therefore propose that the term Interventional Management (IM) may better describe this therapeutic space. The intention is not to replace established guideline terminology, but to recognize IR as a distinct therapeutic domain capable of complementing, preceding, avoiding, or following surgery. A three-pillar framework may be useful conceptually: NOM for observation and supportive treatment without procedural intervention; IM for active image-guided treatment; and OM for surgical control. These categories are not mutually exclusive. The same patient may move between them or receive different strategies sequentially within a single resuscitation pathway.

Within this framework, Damage Control Interventional Radiology (DCIR) represents perhaps the clearest expression of the changing role of IR in acute care. First introduced within the PRESTO paradigm as a time-conscious approach for severely bleeding trauma patients (3), DCIR applies damage-control principles to endovascular treatment by emphasizing early activation, rapid identification of the dominant bleeding source, simplified, effective hemostatic techniques, and prioritizing physiological stabilization over anatomical perfection. More recently, Ascenti et al. described DCIR as a bridge between NOM and damage control surgery and questioned its classification as simply another component of NOM (4). This distinction is more than semantic. It places the interventional radiologist within the early treatment strategy alongside emergency physicians, trauma surgeons, anesthesiologists, and intensivists, rather than at the end of an escalation pathway after conservative management has failed. The key question is therefore no longer simply “surgery or no surgery?”, but which combination and sequence of supportive, interventional, and operative strategies can achieve the safest and fastest control of a life-threatening process.

Several contributions to this Research Topic illustrate how endovascular treatment is shifting from rescue therapy to earlier, more strategically timed interventions. Martinez et al. challenge the persistent perception of embolization as a last-resort option for posterior epistaxis, emphasizing its feasibility and safety, as well as the need for meticulous angiographic assessment given complex vascular anatomy and potentially dangerous extracranial-intracranial and ophthalmic anastomoses. Zhong et al., in 116 patients with non-variceal gastrointestinal bleeding, compare selective and super-selective transcatheter arterial embolization. In upper gastrointestinal bleeding, super-selective embolization was associated with a lower rate of early rebleeding, whereas the lower gastrointestinal subgroup highlighted the ongoing importance of ischemic risk and embolic agent selection. These findings reinforce a central principle of emergency IR: success cannot be reduced to arterial occlusion alone, but requires hemostasis at the appropriate anatomical level while preserving viable tissue whenever possible.

The timing and intent of embolization are becoming equally important. Tiralongo et al. examine prophylactic transarterial embolization after successful endoscopic hemostasis in high-risk non-variceal upper gastrointestinal bleeding. Although the available evidence does not support routine prophylactic embolization, selected patients with unfavorable clinical and anatomical characteristics may benefit from an early strategy aimed at preventing rebleeding and reducing the need for surgical rescue. A comparable preventive concept is explored by Wang et al. in selected patients with small acute epidural hematomas and skull fractures involving meningeal vascular structures. All patients had angiographically demonstrated middle meningeal artery injury; following embolization, no hematoma expansion was observed and craniotomy was avoided. In both settings, IR was deployed before clinical failure rather than in response to it. This shift from reactive rescue toward risk-adapted preventive intervention may become increasingly relevant as patient selection improves.

Acute traumatic and iatrogenic hemorrhage remains one of the clearest expressions of emergency IR. Garducci et al. report transcatheter arterial embolization with n-butyl cyanoacrylate metacryloxysulfolane in 54 patients with traumatic or iatrogenic non-visceral arterial bleeding. Technical success was achieved in all procedures and clinical success in 85.2%, with no procedure-related deaths, major complications, or off-target embolization. Beyond the specific embolic agent, the study reflects the practical demands of emergency embolization: familiarity with different materials, rapid adaptation to vascular anatomy and flow, management of coagulopathy, and the ability to balance selectivity against the urgency of hemorrhage control. From a damage-control perspective, technical sophistication remains important, but should not delay effective hemostasis.

A similar balance between anatomy, timing, and treatment burden emerges from the neurovascular contributions. Quiceno-Ramírez et al. compare endovascular coiling with surgical clipping for ruptured intracranial aneurysms in a real-world cohort. Hierarchical analysis favored coiling for in-hospital outcomes, although attenuation after multivariable adjustment appropriately emphasizes the importance of patient selection and cautions against interpreting the results as evidence of intrinsic superiority. Peña Giraldo et al. describe mechanical thrombectomy with a NeVa stent retriever in severe cerebral venous sinus thrombosis associated with neurological deterioration, achieving near-complete recanalization and favorable short-term functional recovery. Sun et al. evaluate the Lattice flow diverter with mechanical balloons for unruptured intracranial aneurysms, reporting high procedural success and favorable follow-up outcomes. Although elective aneurysm treatment falls outside a strict definition of emergency IR, advances in controlled deployment, wall apposition, device technology, and the management of complex vascular anatomy expand the technical capabilities available when similar challenges arise under urgent conditions.

Emergency IR also extends beyond treatment of the primary disease to the prevention and management of procedural complications. Cheng et al. describe percutaneous thrombin injection with intra-arterial balloon protection for iatrogenic puncture-site pseudoaneurysms, achieving sac thrombosis while preserving the parent artery in their small series. The technique may be particularly useful when antiplatelet therapy cannot be interrupted and illustrates how percutaneous and endovascular approaches can be combined to avoid open repair. Juan-Salvadores et al., in a prospective real-world cohort of 597 patients undergoing radial catheterization, compare two hemostasis strategies and report shorter compression times and lower rates of mild bleeding, pain, paresis, and hematoma with VIRUNDA®, without a significant increase in immediate radial artery occlusion. Procedural success, therefore, extends beyond completion of the catheter-based treatment to include access-site preservation, patient comfort, and efficient post-procedural care.

Rare but potentially severe complications further illustrate the breadth of expertise required in contemporary interventional practice. Feng et al. describe systemic gas embolism during CT-guided lung puncture under general anesthesia, including severe cardiovascular and neurological presentations, and emphasize immediate recognition, oxygen administration, procedural precautions, and multidisciplinary coordination. Xu and Xia report cerebral lipiodol embolism following transarterial chemoembolization for hepatocellular carcinoma, drawing attention to high-risk vascular pathways and delayed neurological manifestations. Corvino et al. review immediate, early, and late complications of tunneled hemodialysis catheters, focusing on image guidance, asepsis, operator expertise, and standardized post-placement management. Wang et al. address the difficult balance between bleeding and thrombosis in patients receiving antithrombotic therapy who undergo synchronous lung biopsy and microwave ablation. Collectively, these studies broaden the concept of emergency IR from the treatment of an acute condition to the recognition, prevention, and management of complications arising from increasingly complex minimally invasive care.

Organ preservation provides another link between several contributions. Salinas-Castro et al. report partial splenic embolization for portal hypertension-related hypersplenism in pediatric patients, with hematological improvement, preservation of splenic tissue, and no major adverse events in their series. Oh et al. investigate splenic infarction and long-term remodeling after splenic artery aneurysm embolization and identify post-embolization splenic staining as a predictor of infarction and subsequent regeneration. These findings illustrate an increasingly important principle of contemporary embolization: the desired endpoint is not necessarily maximal devascularization, but a calibrated intervention that achieves clinical efficacy while preserving organ function. Luo et al. approach personalization from another perspective by developing a nomogram to predict recurrence after percutaneous transforaminal endoscopic decompression. Although different from the acute vascular scenarios represented elsewhere in the Research Topic, this study reflects the broader movement toward individualized risk estimation, more informed patient selection, and tailored surveillance in minimally invasive treatment.

The evolution of emergency IR, however, cannot depend on technical progress alone. The recently published CIRSE Standards of Practice for the Provision of Emergency IR Care on a 24/7 Basis recognize emergency IR as part of direct clinical care and emphasize sustainable round-the-clock availability, written multidisciplinary pathways, adequate staffing and infrastructure, uninterrupted access to cross-sectional imaging and an IR suite, and measurable key performance indicators such as CT-to-needle and door-to-treatment-decision (5). These recommendations closely align with the broader message emerging from this Research Topic. If an interventional procedure is to serve as a genuine treatment option rather than a last-resort rescue, the service must be available when the clinical decision is made. Delayed access may effectively remove IR from the therapeutic algorithm regardless of its technical efficacy.

In this context, time deserves greater attention as an outcome of emergency IR. Consultation-to-puncture, CT-to-needle, and time-to-hemostasis may provide meaningful measures of performance alongside survival, rebleeding, transfusion requirements, organ preservation, ischemic complications, functional recovery, rescue surgery, and access-site outcomes. Evaluating these endpoints will require prospective registries, multicenter comparative studies, pragmatic trials, and more standardized reporting. Institutional pathways should also allow IR involvement before deterioration occurs, with clear interfaces between interventional radiology, surgery, endoscopy, anesthesia, intensive care, and emergency medicine. Sustainable 24/7 staffing, rapid access to CT and angiography, adequate device inventories, and hybrid environments where available are therefore not simply organizational details; they determine whether minimally invasive treatment can actually be delivered within the relevant therapeutic window.

The 17 contributions collected in this Research Topic ultimately describe a specialty whose role in the emergency pathway is becoming increasingly difficult to define as merely “non-operative.” IR may provide definitive hemostasis, prevent anticipated deterioration, replace surgery in selected patients, complement an operation, manage an iatrogenic complication, or stabilize physiology before definitive treatment. DCIR describes this role particularly well in the most time-critical hemorrhagic scenarios. At the same time, Interventional Management may offer a broader framework for understanding the place of image-guided therapy alongside NOM and OM. The value of such terminology will depend on whether it improves clinical thinking rather than simply adding another label. Its purpose is to emphasize that interventional radiologists should be involved in formulating treatment strategies, not only when a procedure has already been requested. The future of emergency IR will therefore depend on technical innovation, but equally on earlier integration, stronger evidence, shared clinical pathways, sustainable 24/7 services, and recognition of the interventional radiologist as a clinician within the emergency treatment team.
